# Pump up the volume

**DOI:** 10.7554/eLife.100032

**Published:** 2024-07-02

**Authors:** Qin Ni, Sean X Sun

**Affiliations:** 1 Institute for NanoBioTechnology and Department of Mechanical Engineering, Johns Hopkins University Baltimore United States

**Keywords:** cell migration, neutrophil, cell size, cell volume, physical forces, Human

## Abstract

An influx of water molecules can help immune cells called neutrophils to move to where they are needed in the body.

**Related research article** Nagy TL, Strickland E, Weiner OD. 2024. Neutrophils actively swell to potentiate rapid migration. *eLife* **13**:RP90551. doi: 10.7554/eLife.90551.

Cell migration is essential for most processes in the body, such as embryonic development, immune responses or wound repair. The ability of cells to migrate is a complex phenomenon that depends on many factors, including the polymerization of actin filaments. For a cell to move, actin filaments and other proteins in the cytoskeleton actively polymerize against the cell membrane to generate protrusions and push the cell surface forward.

In recent years, however, it has become evident that ion and water flow through the cell membrane might also be involved in cell movement (Stroka et al., 2014; Li et al., 2020). A polarized distribution of ion channels and transporters can generate a small osmolarity gradient in the cytoplasm, and the resulting influx of ions and water molecules can cause the cell to swell at its leading edge and to shrink at its trailing edge, thereby enabling it to move. However, the mechanisms underlying these processes are not fully understood.

Now, in eLife, Tamas Nagy, Evelyn Strickland and Orion Weiner at the University of California San Francisco report that innate immune cells known as neutrophils rely on water flux to help them move quickly to the sites of infection or injury (Nagy et al., 2024). It is well known that neutrophils use actin to relocate, but it has also been shown that they can increase the influx of water into the cell – and consequently their volume and motility – in response to molecules known as chemoattractants (Weiner et al., 1999).

To better understand the impact of water and ions on cell movement, Nagy et al. used a technique, called Fluorescence Exclusion Method, which can track changes in the volumes of single cells (Cadart et al., 2017). This revealed that when the neutrophils were exposed to chemoattractants, the cells started to swell and became mobile.

Building on these findings, Nagy et al. sought to identify the molecules that regulate water-driven migration in neutrophils: this was a challenging task because numerous ion transporters and regulatory proteins are involved in the process (Hoffmann et al., 2009). To navigate this complexity, they combined genome-wide CRISPR/Cas9 knockout screening with an approach that separated cells according to their buoyant density (Shalem et al., 2014). In brief, they generated single-gene knockouts for every gene in the genome and identified which knockouts swelled when exposed to chemoattractants, and which did not.

Many of the genes they identified as being involved in cell swelling and migration were already known, such as the genes encoding the ion transporters NHE1 and AE2 (Li et al., 2021). However, a gene called PI3Kγ – primarily known for its roles in cell growth and cell phenotype specification – also emerged as a candidate (Mendoza et al., 2011; Madsen, 2020). Nagy et al. then performed further experiments to confirm that these regulators were responsible for the positive correlation between the changes in cell volume and migration velocity. Tests involving hypo-osmotic shocks provided evidence of the relationship between NHE1-driven water flux and cell motility. In summary, Nagy et al. demonstrated that cell swelling is both necessary and sufficient for neutrophils to move following stimulation with chemoattractants. It also complements cytoskeletal rearrangements to enhance migration speed.

The exciting findings by Nagy et al. usher in a new era of exploration that intersects cytoskeletal dynamics and cell electrophysiology. Intracellular electrophysiological homeostasis – the balance between ions, proteins and water – is maintained by a complex system involving numerous ion transporters and the actin cytoskeleton.

Conversely, the intracellular ionic environment can influence cytoskeletal activity and force generation (de Boer et al., 2023; Webb et al., 2011). Previous studies have shown that molecular interactions between actin, NHE1 and Akt (which is a target of PI3K) regulate actin organization, intracellular pH and cell migration, while recent work suggests that non-cancerous cells often use actin-NHE1 crosstalk to mediate mechanosensitive adaptations to environmental stimuli (Denker et al., 2000; Denker and Barber, 2002; Meima et al., 2009; Ni et al., 2024).

Collectively, these studies highlight a deeply interconnected system where PI3K and Akt form a hub that potentially links the mechanical ‘players’ in the cell (such as F-actin and myosin II) with the electrophysiological players (such as NHE1)(De Belly et al., 2023), thereby regulating cell migration, mechanosensation and growth.

Given the complicated nature of this mechano-electrophysiological system, comprehensive, high-throughput methods (such as genome-wide knockout screening) are highly valuable. Mathematical models based on such large-scale data will also be instrumental in understanding the underlying interactions. For example, large-scale genomic datasets have revealed the importance of ion transporters in cancer cells, with key elements once again being ion transporters in the NHE and AE families (Shorthouse et al., 2018). It is likely that the system governing ionic and water content regulation, cell migration and metabolism forms the basis of essential biological processes such as growth and morphogenesis, and that alterations in the system could be the origin of many diseases.
